# Evaluation of proton beam radiation-induced skin injury in a murine model using a clinical SOBP

**DOI:** 10.1371/journal.pone.0233258

**Published:** 2020-05-22

**Authors:** Pietro Pisciotta, Angelita Costantino, Francesco Paolo Cammarata, Filippo Torrisi, Giovanna Calabrese, Valentina Marchese, Giuseppe Antonio Pablo Cirrone, Giada Petringa, Giusi Irma Forte, Luigi Minafra, Valentina Bravatà, Massimo Gulisano, Fabrizio Scopelliti, Francesco Tommasino, Emanuele Scifoni, Giacomo Cuttone, Massimo Ippolito, Rosalba Parenti, Giorgio Russo

**Affiliations:** 1 Physics and Astronomy Department, University of Catania, Catania, Italy; 2 Institute of Molecular Bioimaging and Physiology (IBFM-CNR), Cefalù (PA), Italy; 3 National Laboratory of South, National Institute for Nuclear Physics (LNS-INFN), Catania, Italy; 4 Laboratory of Molecular and Cellular Physiology, Biomedical and Biotechnological Sciences Department, University of Catania, Catania, Italy; 5 Centre for Advanced Preclinical *in vivo* Research (CAPiR), University of Catania, Catania, Italy; 6 Laboratory of Synthetic and Systems Biology, Drug Science Department, University of Catania, Catania, Italy; 7 Molecular Preclinical and Translational Imaging Research Center (IMPRonTe), University of Catania, Catania, Italy; 8 Radiopharmacy Laboratory Nuclear Medicine Department, Cannizzaro Hospital, Catania, Italy; 9 Department of Physics, University of Trento, Povo, Italy; 10 Trento Institute for Fundamental Physics and Applications (TIFPA), National Institute for Nuclear Physics, INFN, Povo, Italy; 11 Nuclear Medicine Department, Cannizzaro Hospital, Catania, Italy; Northwestern University Feinberg School of Medicine, UNITED STATES

## Abstract

The purpose of this paper is to characterize the skin deterministic damage due to the effect of proton beam irradiation in mice occurred during a long-term observational experiment. This study was initially defined to evaluate the insurgence of myelopathy irradiating spinal cords with the distal part of a Spread-out Bragg peak (SOBP). To the best of our knowledge, no study has been conducted highlighting high grades of skin injury at the dose used in this paper. Nevertheless these effects occurred. In this regard, the experimental evidence of significant insurgence of skin injury induced by protons using a SOBP configuration will be shown. Skin damages were classified into six scores (from 0 to 5) according to the severity of the injuries and correlated to ED50 (i.e. the radiation dose at which 50% of animals show a specific score) at 40 days post-irradiation (d.p.i.). The effects of radiation on the overall animal wellbeing have been also monitored and the severity of radiation-induced skin injuries was observed and quantified up to 40 d.p.i.

## 1. Introduction

In recent years, the use of proton therapy is emerging as an alternative to conventional radiotherapy with photons. This is mainly due to the presence of the Bragg peak (BP), which allows higher dose conformation to tumor regions while sparing the surrounding organs reducing side effects [1]. Consequently, the number of ion therapy centres is increasing every year all over the world and, only in the last five years, 42 new particle therapy facilities have started their treatment operations (35 new proton therapy centres and 7 new carbon ion therapy centres) [2].

Several *in vivo* preclinical studies have been performed to evaluate deterministic damages caused by proton therapy paying attention on the doses at which these injuries occur [3–10], but available data are still partial and far to be complete. Indeed, due to several difficulties in performing such kind of studies, robust and reproducible preclinical *in vivo* data are limited to a few studies, mainly performed in the middle of a spread-out Bragg peak (SOBP) [7–10].

The research shown in this paper was originally designed to perform a long observational study investigating the dose at which 50% of small animals develop myelopathy when the spinal cords were irradiated in the distal part of a SOBP. For this purpose, the animals were irradiated with a range of proton doses at spinal cord level to evaluate a possible onset of proton-induced myelopathy. The proton irradiation doses have been chosen taking into account a work published by Yeh Chi Lo *et al*. in which the spinal cords of mice were irradiated with a range of X-ray doses (from 16 to 26 Gy) [11]. Since the doses were high and released in just one fraction, the estimated proton RBE used to re-scale the X-ray doses to protons was supposed to be 1.2 (6). To our knowledge, no study in literature was found that takes into account the characterization of skin injury in the area of treatment used in this paper. Even in Yeh Chi Lo et al. [11], there is no mention of possible skin injury damage. Previous studies described only mild skin injury effects, such as loss of fur, swelling and redness, only at doses higher than the ones used in this study [3,11,12]. For this purpose, this paper wants to highlight the experimental evidence of significant insurgence of skin injury induced by protons through an observational study and how the results could be important for future RBE studies.

In agreement with these assumptions, in this paper a skin deterministic damage has been adopted as endpoint in order to assess the biological impact at the last few mm of a proton SOBP. Healthy mice were irradiated using a range of proton doses to evaluate radiation-induced skin deterministic damage, as well as to verify their possible involvement to animal wellbeing.

## 2. Methods

### a. Ethics statement

The experiments were performed in accordance with the European Communities Council directive and Italian regulations (EEC Council 2010/63/EU and Italian D.Lgs. 26/2014). The project was approved by Italian Ministry of Health (n. 248/2018-PR of 30/03/2018).

Efforts were employed to replace, reduce and refine the use of laboratory animals. To avoid irrelevant suffering to treated mice, euthanasia was performed as soon as the final score was reached. All reasonable efforts were made to reduce suffering, avoiding the most painful procedures. The endpoint used to determine if animals should undergo to euthanasia was reached when lesions showed dimension higher than 0.5 cm.

### b. Animal model

All experiments were performed on 6 weeks old C57BL/6 male mice (Charles River Laboratory), weighing 27 ± 3 gr. Animals were housed in IVC-cages for 9 weeks using a stocking density of maximum 4 mice per cage. In order to identify mice stored within the same cage, the animals were randomly marked on the tail as follows: mouse 1 = NT (No Tag); mouse 2 = 1 tag; mouse 3 = 2 tags; mouse 4 = 3 tags. Animals were fed ad libitum and maintained in the same room under a 12:12-hour light/dark photoperiod at 24°C. To minimize suffering and distress of mice, standard environmental enrichment of nest paper, a cardboard fun tunnel and one wooden chew block were provided.

A total number of 40 animals was used in this study. Mice were randomly assigned to sham-control group (n = 8) and treated group (n = 32). The treated groups were randomly divided into 4 subgroups as follows: Dose 12 Gy (n = 8); Dose 15 Gy (n = 8); Dose 17 Gy (n = 8) and Dose 19 Gy (n = 8). Before each irradiation, the mice were anesthetized with Zoletil (tiletamine) 40 mg/kg and Sedastart (medetomidine) 50 μg/kg and shaved in the treatment region. Animal health and behavior were monitored twice a week. All animals were scanned with an Albira Si microPET/CT to define the irradiation setup as it will be described later on this paper.

### c. Irradiation procedures and dosimetry

#### i. CATANA experimental room and dose delivery system

**Fig 1:**
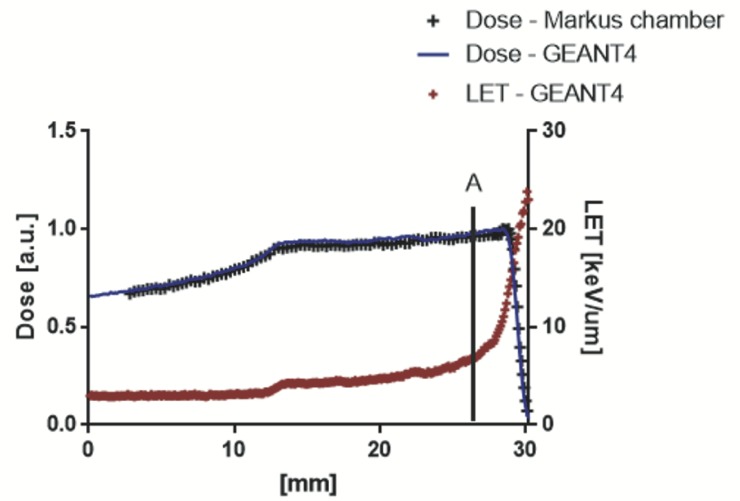
Experimental proton beam configuration: Dose and LET_d_ distribution. The A indicates the skin position along SOBP.

**Fig 2:**
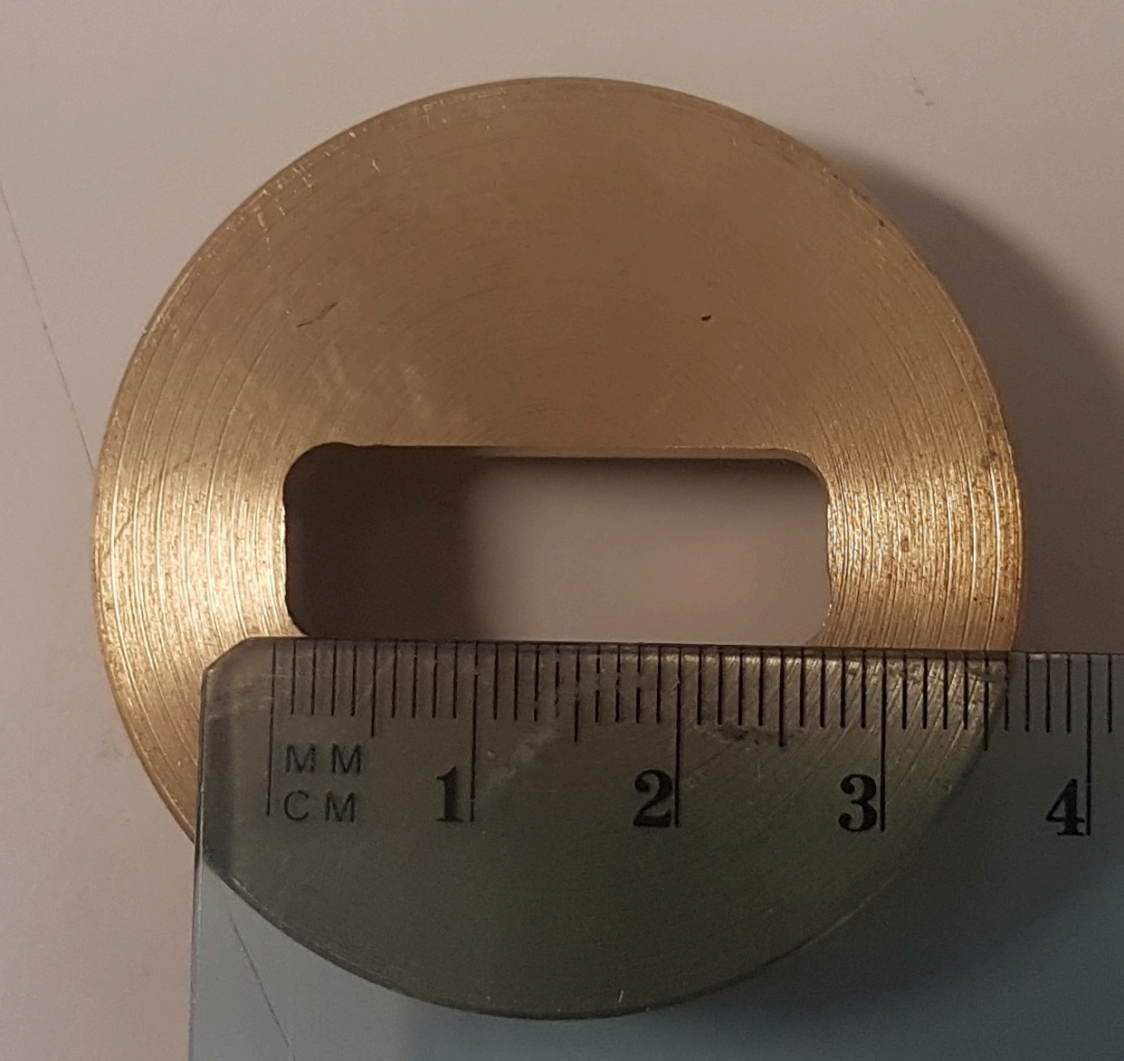
Brass collimator used for irradiation.

All proton treatments were performed at the INFN-LNS CATANA proton therapy facility in Catania (Italy) using a passive proton beam line. Since 2002, CATANA is a proton therapy facility where radiotherapeutic treatments of eye tumours are performed. In this experimental hall, a fixed horizontal beam line is installed and clinical proton beams can be delivered with a maximum energy of 62 MeV. A beam shaping system is used to obtain a uniform dose distribution at the isocenter. Indeed, when the proton beam reaches the experimental hall, it goes out in air and flies for three meters before hitting the target. Along its path, the beam is intercepted by various elements in order to obtain flat transversal dose distribution at the isocenter, as described elsewhere [1,13,14]. A dedicated positioning animal holder system (1) was used to ensure a precise and reproducible positioning. The lateral beam inhomogeneity was less than 2% at irradiation isocenter and, before irradiation, a check of beam flatness was performed using a motorized silicon detector. A PMMA modulator wheel was used to create a SOBP featured by a plateau of 14.8 mm and a practical range of 30.2 mm. Moreover, a range shifter was used to place the animal skin at the desired position as shown in Fig 1. The uncertainty in the determination of the absorbed dose during the beam calibration process was evaluated to be less than 3%. The field size was shaped using a 6.3 mm thick brass in-house-made collimator with a rectangular opening of 10 x 25 mm (see. Fig 2).

The dosimetry was performed using a Markus ionization chamber (PTW Freiburg GmbH, Germany) and Gafchromic EBT3 films (ISP Corp., New York, USA). During each irradiation, a Gafchromic EBT3 film was placed just before the target to check the beam flatness and the released dose. The films were scanned 24 hours after irradiation with an Epson Expression 10000 XL Scanner (Epson, Germany) and analysed by homemade Matlab^TM^ script. The dose delivery was monitored by a transmission ionization chamber placed along the beam line that automatically switched off the beam when the requested number of monitor units (MU) were reached. The calibration of MU as a function of the absolute dose to water was determined by measurements with the Advanced Markus IC at middle SOBP position. A constant dose rate of 5 Gy/min was set for all the irradiations. All animal irradiations were performed at the same time interval of the day to avoid differences related to circadian rhythm of mice.

#### ii. Dosimetric evaluation using GEANT4 toolkit

Monte Carlo (MC) simulation is usually applied for routine clinical applications as it allows to perform accurate and efficient prediction of dose distribution inside the target. Moreover, when small animals are irradiated, the power of this tool becomes more important because the target dimensions are less than a few centimetres. The application developed by our group allows the simulation of *in vivo* experiments performed at the CATANA experimental room including the ability to implement the target through its DICOM microCT images [13]. Specifically, the use of this application makes the simulation of beam interaction with real geometry of small animals possible. The physics list used for the simulations includes the *QGSP_BIC* models and *G4EmStandardPhysics option4*, respectively, for the hadronic and electromagnetic processes. In particular, the first one is recommended for proton energies below 200 MeV/A and includes the binary cascade model. The *option4* of the Electromagnetic (EM) Standard GEANT4 physics is a physics constructor designed for any applications requiring higher accuracy of electrons, hadrons and ion tracking. It uses the most accurate standard and low-energy models and a set of EM processes with accurate simulations of neutral and charged particle transport. Both *QGSP_BIC* models and *G4EmStandardPhysics option4* have been widely validated for, among other particles, proton incident beams at the energy of interest for medical applications [15–17].

**Fig 3:**
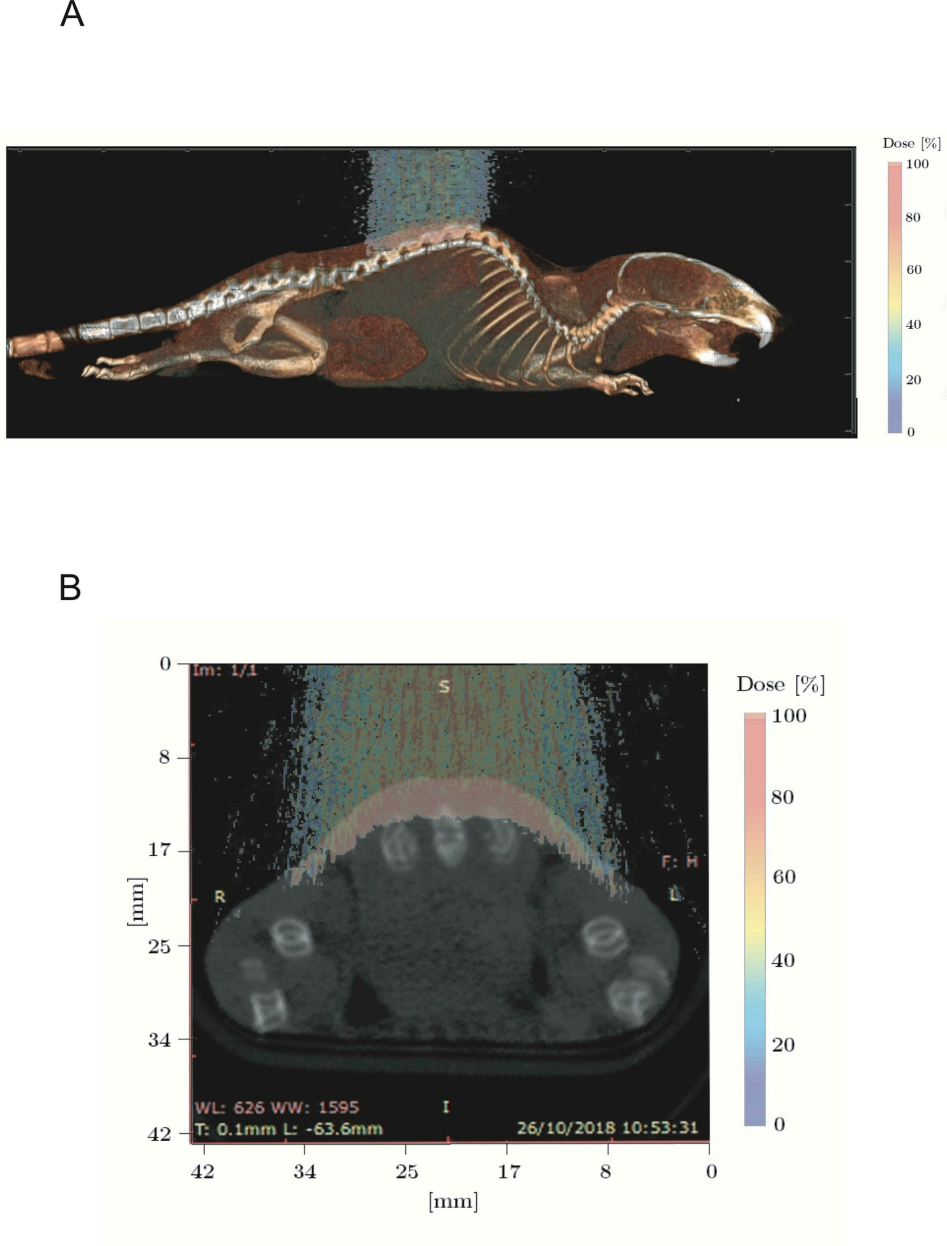
Dose distribution obtained using GEANT4-based application. **A)** coronal dose distribution; **B)** axial dose distribution.

The GEANT4-based application was used to define the irradiation setup and in detail to determine which combination of modulator wheel and range shifter was suitable to correctly position the target along the SOBP. In addition, it was used to calculate the dose distributions and the LET_d_ value occurring by SOBP in the target. Indeed, our GEANT4 application permits to simulate entirely the CATANA proton beamline and defines voxel-by-voxel the real composition of the target using the real DICOM micro-CT [1,13,14]. The micro-CT datasets were acquired using a preclinical micro-PET/CT (Albira Si, Bruker) available at CAPiR (Centre for Advanced Preclinical *in vivo* Research), University of Catania, Italy. Datasets were acquired using 600 views in high resolution configuration, an x-ray energy of 45 kVp, a current of 400 μA and the dimension of each CT-voxel was equal to 125 x 125 x 125 μm^3^. A treatment plan simulation was performed for each mouse, as shown in Fig 3, in order to evaluate if some OAR (e.g. lungs) could receive dose and, in the case, to modify the treatment accordingly, using a trial-error method.

### d. Skin injury score assignment

Proton-induced skin injuries were evaluated paying attention to erythema, microlesions and ulcers. To monitor the skin alterations, mice were weighed and photographed once a week. The mice pictures acquired once a week were grouped per dose level and ordered in a photographic time-lapse. The assignment of the skin injury score was carried out during the check and independently confirmed using recorded images.

According to a modified scoring system [18], our scoring classification was defined as follows:

- SCORE 0 = healthy skin;
- SCORE 1 = light redness;
- SCORE 2 = redness, erythema principle, alopecia;
- SCORE 3 = extended microlesions, early-stage ulcers);
- SCORE 4 = confluent moist desquamation, ulcers;
- SCORE 5 = open wound, necrosis.

Skin injury was estimated by a semi-quantitative scale and mean score was calculated at different time-points (7, 14, 21, 30 and 40 d.p.i.) for each treatment group.

Body weight changes were quantified as a function of dose considering the mean weight of each treatment group compared to the control group.

The impact of skin injury progression on mice survival was assessed calculating survival curves using GraphPad Software version 5 (GraphPad Software, Inc.). This software uses the product limit method of Kaplan and Meier and compares survival curves using both the logrank test and the Gehan-Wilcoxon test. Survival curves were plotted as a function of time (7, 14, 21, 30 and 40 d.p.i.) classifying the animals as follows: “1” mouse deceased at a certain time-point and “0” if it survived until the end of the observation time (40 d.p.i.).

### e. Statistical analysis

All statistical results were plotted using GraphPad Prism 5. The median skin injury score was calculated for each experimental group and the statistically significant differences between irradiated and control groups were analysed using one-way analysis of variance (ANOVA).

Bonferroni and Holm multiple comparison method was used to evaluate the differences between experimental groups as a *post hoc* test when the ANOVA reported statistically significant differences. All statistical tests were performed by p-values (p<0.05 was considered statistically significant). The number of asterisks shows the statistical significance and are correlated with the p-value: *p<0.05, **p<0.01. Non-parametric methods were used for the skin injury scores because the scoring system is ordinal, not interval.

## 3. Results

### a. MC: Depth dose profile and LET assessment

Specific dosimetric evaluations were computed for each animal used in this work thanks to the use of our GEANT4-based application. In detail, it allowed to define the best irradiation configuration and to calculate the dose distributions and the mean total LET value occurring by SOBP on skin. The mean LET value calculated at skin surface was equal to 6.68 keV/μm. In Fig 3A and 3B, an example of dose distribution calculated by our application is shown.

### b. Skin injury evaluations

Four groups of mice were irradiated with a range of proton absorbed dose (12, 15, 17 and 19 GyRBE) and one group was used as a negative control (sham-CTRL). As expected, the monitoring of mice has highlighted a dose-dependent relationship between the onset of skin damage and the amount of radiation dose delivered.

**Fig 4:**
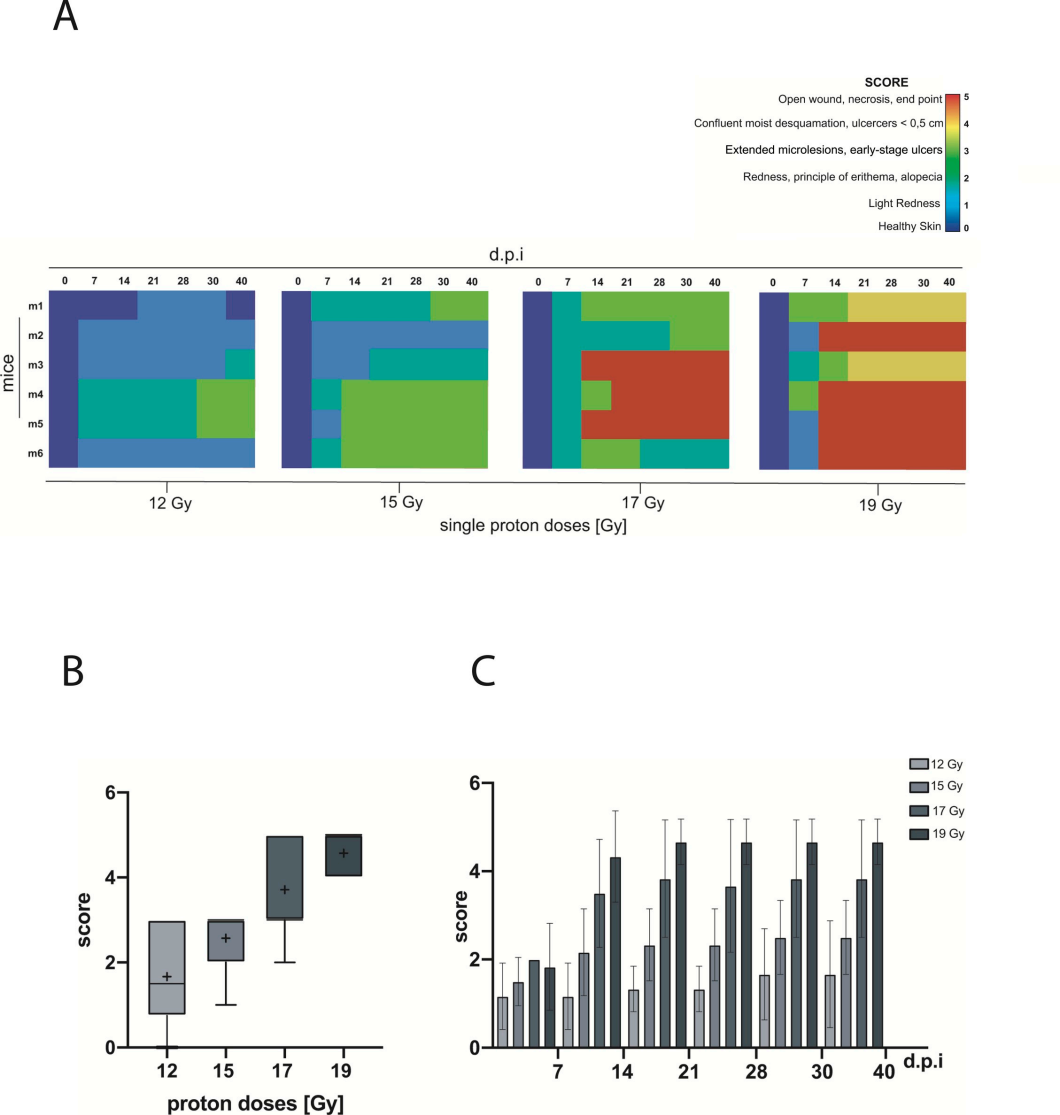
Proton beam-induced skin injury. **A)** Qualitative “heat map” for single irradiated groups at 7, 14, 21, 28, 30 and 40 d.p.i.. **B)** Box plot: skin injury score as a function of dose at 40 d.p.i.. The mean score is represented by the plus sign (+). **C)** Skin injury score progress analysis at 7, 14, 21, 28, 30 and 40 d.p.i for single dose (12, 15, 17 and 19 Gy).

A qualitative heat map per dose is shown in Fig 4A, where each row corresponds to a single mouse and each column corresponds to a day of observation.

Mice exposed to 12 Gy developed first symptoms of skin damage at 7 d.p.i. as shown in Fig 4A where a small increase in colour nuance in 5/6 blocks is reported. In details, 50% of the animals showed the first skin injury symptoms with light redness (SCORE 1) while 33% showed redness, principle of erythema and/or alopecia (SCORE 2).

This condition evolved in extended micro-lesions (SCORE 3) at 30 d.p.i only in 33.3% of animals, but without reaching the SCORE 5. Additionally, a complete recovery was registered in 16.7% of the animals.

Skin injuries induced by 15 Gy arose with SCORE 2 at 7 d.p.i in 50.0% of the animals, as shown in Fig 4A. This condition evolved into the next level of severity (SCORE 3) at 14 d.p.i in 50% and at 30 d.p.i in 66.6% of mice.

Qualitative heat map of mice exposed to 17 Gy (see Fig 4A) showed the first symptoms of skin injury (SCORE 2) at 7 d.p.i.. Then, 50% of mice evolved from SCORE 2 to SCORE 3 at 14 d.p.i. and 33.3% of mice were characterized by open-wounds and necrosis (SCORE 5) that lead to their sacrifice before the last time-point (40 d.p.i.). After 21 d.p.i., a partial recovery in 33.3% of these animals from SCORE 3 to SCORE 2 was observed, characterized by an alopecia area corresponding to the irradiation site.

Moreover, the mice irradiated with the highest proton dose (19 Gy) showed the first sign of skin injury at 7 d.p.i.. The corresponding qualitative heat map (see Fig 4A) shows that skin lesions originate with the lowest degree of severity (SCORE 1) in 50% of animals, but degenerates quickly reaching the final score (SCORE 5) at 14 d.p.i. in 66.6% of the cases.

The box plot in Fig 4B shows the skin injury score as a function of dose at the end of observation (40 d.p.i.). The mean score per dose was represented by the plus sign (+) and the corresponding score values are shown in Table 1. The box plot (Fig 4B) shows also median values per single proton beam dose (see Table 1).

**Table 1:** Skin injury score values per dose.

SCORE	12 Gy	15 Gy	17 Gy	19 Gy
**min**	0	1	2	4
**max**	3	3	5	5
**median**	1.5	3	3	5

Statistical analysis of mean scores was calculated with ANOVA test using Bonferroni and Holm multiple comparison and confirms that first symptoms of skin injury arise at 7 d.p.i for all treatment groups without statistically significant differences among the experimental groups (Fig 4C). Starting from 14 d.p.i. severity of skin damage is characterized by progressive dose-dependent trend.

In addition, no statistically significant differences were found between the doses of 12 and 15 Gy (*p-value < 0.05, Fig 4C). Indeed, despite the lesion progression induced by 15 Gy is faster than 12 Gy, the SCORE 5 was not achieved and the trend of bars corresponding respectively to the dose of 12 and 15 Gy are similar. Furthermore, the progressive trend of the single dose of 19 Gy curve showed no significant differences with 17 Gy group until the end of the monitoring (40 d.p.i.). The statistically significant differences between doses are listed in Table 2.

**Table 2:** Statistical analysis between lower (12–15 Gy) and higher (17–19 Gy) doses.

Dose (Gy)	p-value	T-value	SD
**12 vs 15**	p>0.05 p = 0.3478593	2.0112	0.51639_**(12 Gy)**_ 0.8164 _**(15 Gy)**_
**12 vs 17**	**p<0.01 p = 0.0003867	5.0280	0.51639_**(12 Gy)**_ 1.329 _**(17 Gy)**_
**12 vs 19**	**p<0.01 p = 9.5629e-06	6.7040	0.5164 _**(12 Gy)**_ 0.5163 _**(19 Gy)**_
**15 vs 17**	*p<0.05 p = 0.040872	3.0168	0.8164 _**(15 Gy)**_ 1.3291_**(17 Gy)**_
**15 vs 19**	**p<0.01 p = 0.0008384	4.6928	0.8164 _**(15 Gy)**_ 0.5164 _**(19 Gy)**_
**17 vs 19**	p>0.05 p = 0.6558068	1.6760	1.3291_**(17 Gy)**_ 0.5164 _**(19 Gy)**_

**Fig 5:**
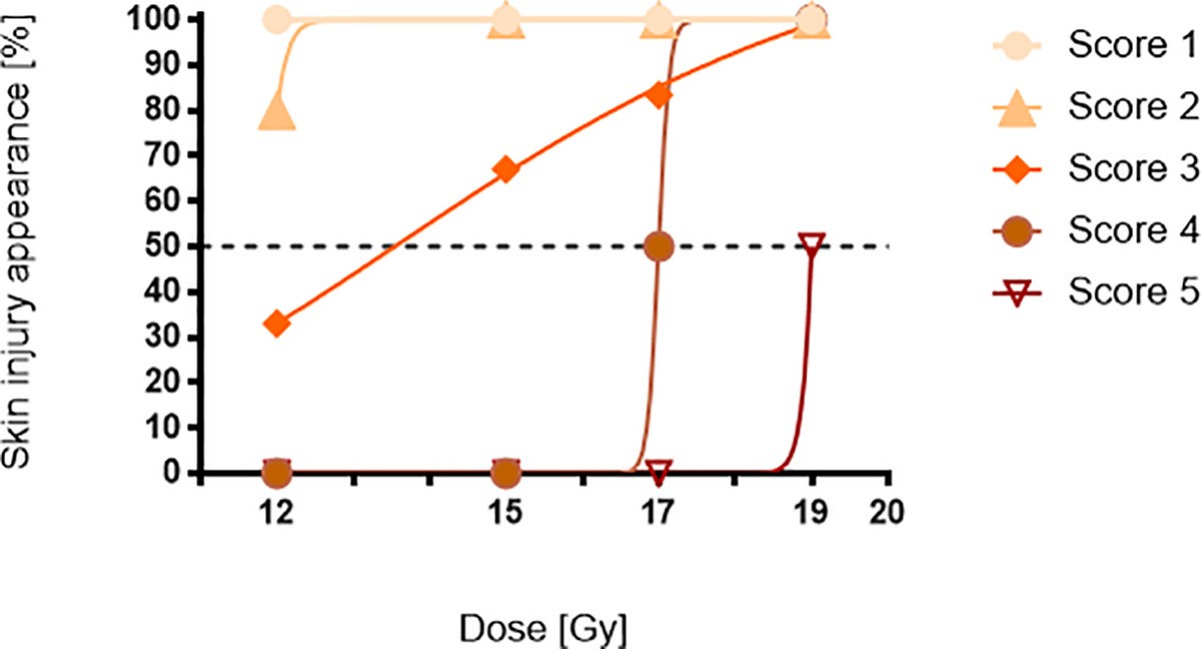
Acute skin damage score results and sigmoidal fit.

The dose–response curves for skin reaction induced by protons is shown in Fig 5. Data presented is the percentage of animals in each treatment group showing a severe reaction, previously described in section 2d, at 40 d.p.i.. The radiation doses at which 50% of animals show a specific score (ED50) were calculated interpolating our data using a sigmoidal fitting curve (Eq 1) and the results are shown in Table 3.

$$Y=\frac{X^{n}}{D^{n}+X^{n}}$$ (Eq 1)

where X is the absorbed dose, Y represents the percentage of skin injury appearance, D is the dose that induces a halfway effect. X and D are raised to the n^th^ power.

**Table 3:** ED50 evaluated at different skin injury score.

SCORE	1	2	3	4	5
**ED50**	NC	NC	13.6 Gy	17 Gy	19 Gy

**Fig 6:**
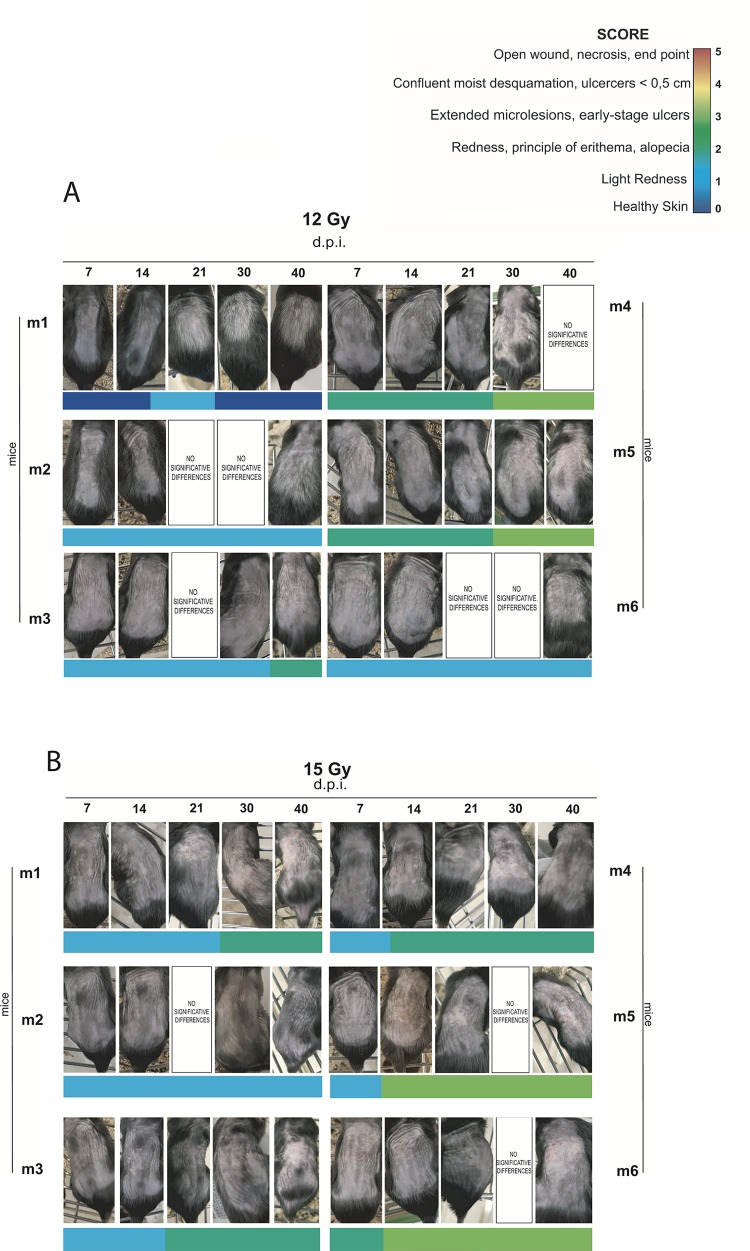
Photographic time-lapse of skin injury progress at 12 and 15 Gy. Panels with lettering “no significant difference” indicated that no significant variations between the previous photo are observed.

**Fig 7:**
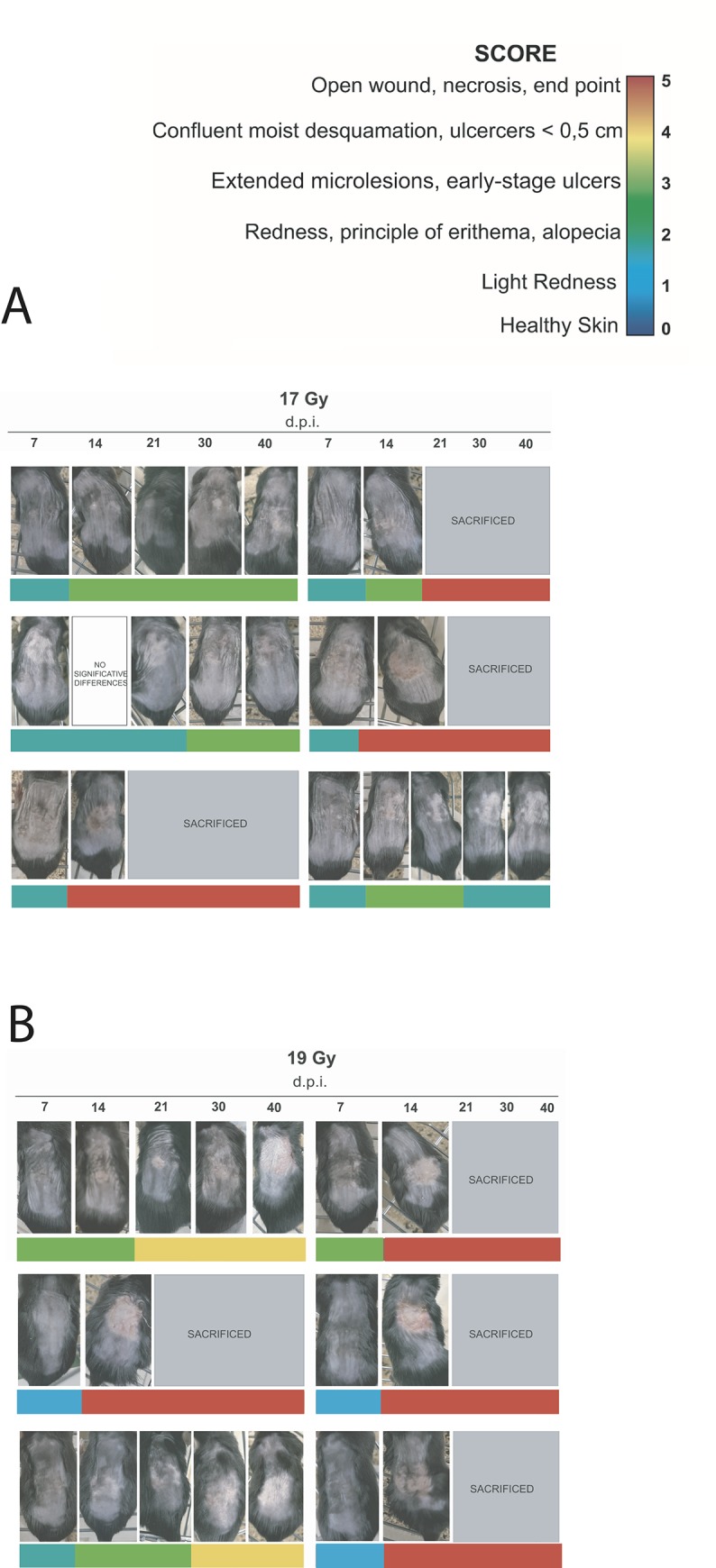
Photographic time-lapse of skin injury progress at 17 and 19 Gy. Panels with lettering “no significant difference” indicated that no significant variations between the previous photo are observed.

The data shown suggest that the onset of the skin injury started just at 7 d.p.i in all treated mice regardless of the single dose received. In detail, the doses of 12 Gy and 15 Gy induced slightly reddened skin (SCORE 1) accompanied, in some cases, by diffuse erythema (SCORE 2). Severer effects were found in the animals irradiated with single doses of 17 Gy and 19 Gy which caused diffuse microlesions (SCORE 3). The photographic time-lapse, shown in Fig 6 and Fig 7, and the qualitative heat map indicated that the progression of the skin injury continues with a dose-dependent high-trend of severity from 14 d.p.i. until the end of the monitoring.

### c. Proton irradiation and overall health conditions of treated mice

Mice body weight changes were monitored once a week in order to evaluate whether the health conditions and the observed increase in lethality were influenced by proton-induced skin injury.

**Fig 8:**
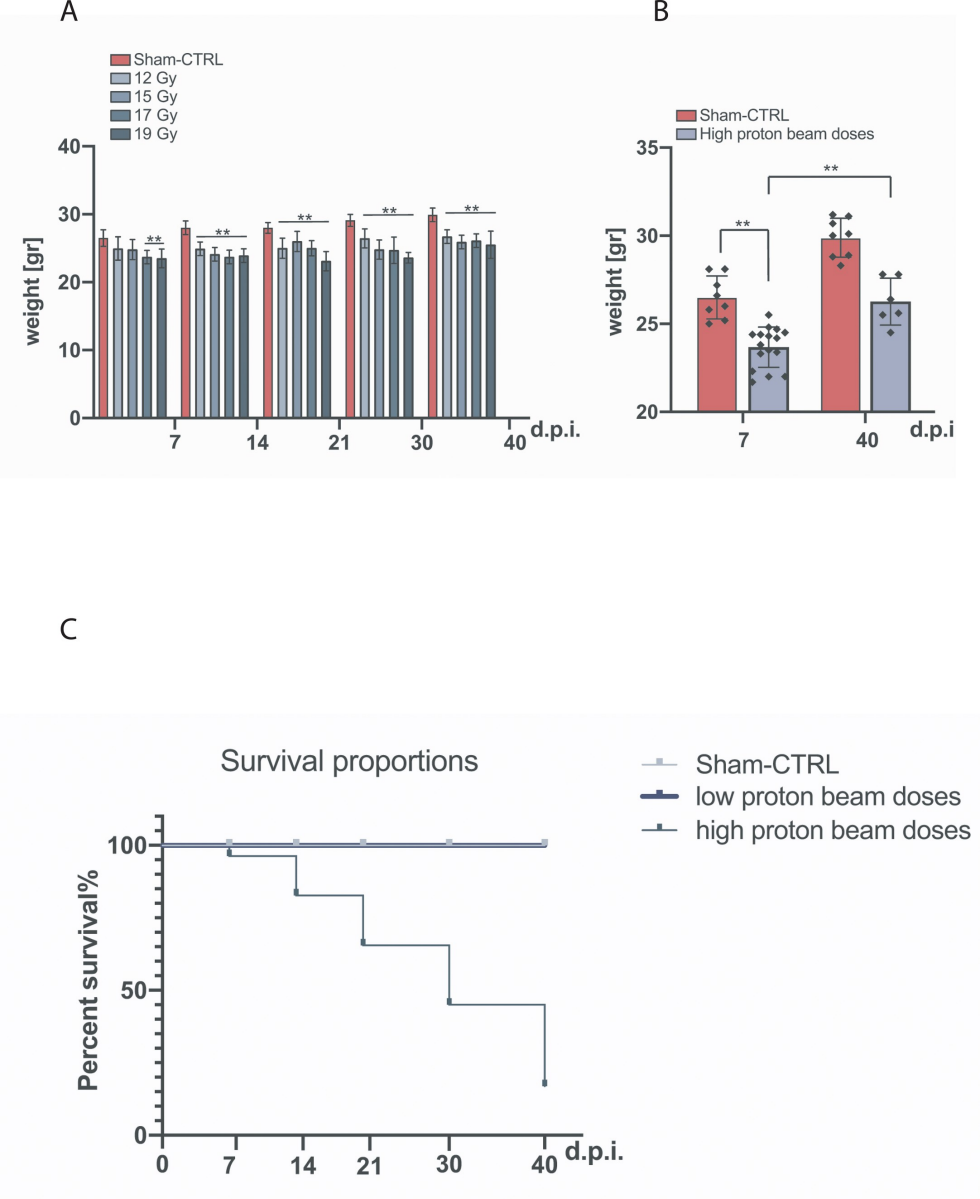
Vitality impairment induced by proton high dose. **A)** Sham-CTRL group and treated group body weight at 7, 14, 21, 30 and 40 d.p.i; **B)** Body weight of mice irradiated at 17 and 19 Gy compared to sham-CTRL group at 7 d.p.i. and 40 d.p.i.. **C)** Survival proportions plot of low and high proton beam doses compared to sham-CTRL.

The mean weight of each treated group is shown in Fig 8A as a function of time and compared to the sham-CTRL group at 7, 14, 21, 30 and 40 d.p.i.. A statistically significant weight loss was observed at each time point for mice irradiated with single dose of 17 Gy and 19 Gy when compared to the sham-CTRL group as confirmed by ANOVA test results shown in Table 4A and Table 4B.

**Table 4:** ANOVA test results of 17 and 19 Gy compared to sham-CTRL group.

**A)**				
	**d.p.i**	**p-value**	**T-value**	**SD**
**17 Gy vs sham-CTRL**	7	**p<0.01 p = 0.024757	4.0800	0.9710 _**(17 Gy)**_ 1.2130 _**(shamCTRL)**_
	14	**p<0.01 p = 3.7860e-06	6.5916	1.1463 _**(17 Gy)**_ 1.2772 _**(shamCTRL)**_
	21	**p<0.01 p = 0.0007880	4.7110	1.1176 _**(17 Gy)**_ 0.7863 _**(shamCTRL)**_
	30	**p<0.01 p = 0.0001608	5.3258	1,9568 _**(17 Gy)**_ 0.8701 _**(shamCTRL)**_
	40	**p<0.01 p = 0.0005106	4.8784	1.3889 _**(17 Gy)**_ 1.1076 _**(shamCTRL)**_
**B)**				
**19 Gy vs sham-CTRL**	7	**p<0.01 p = 0.002019	4.1528	1.3680 _**(19 Gy)**_ 1.2130 _**(shamCTRL)**_
	14	*p<0.05 p = 0.0110938	3.6344	1.4142 _**(19 Gy)**_ 1.2772 _**(shamCTRL)**_
	21	**p<0.01 p = 0.0031802	4.1722	1.4142 _**(19 Gy)**_ 0.7863 _**(shamCTRL)**_
	30	**p<0.01 p = 0.0009967	4.6204	0.7778 _**(19 Gy)**_ 0.8701 _**(shamCTRL)**_
	40	*p<0.05 p = 0.036357	3.2089	1.6263 _**(19 Gy)**_ 1.1076 _**(shamCTRL)**_

On the other hand, statistically significant changes in body weight were observed for mice irradiated with single dose of 12 Gy and 15 Gy when compared to the sham-CTRL group only at 14, 21, 30 and at 40 d.p.i. as confirmed by ANOVA test results shown in Table 5A and Table 5B. Furthermore, no significant differences in weight loss were found among single doses. Interestingly, despite no statistically significant weight changes characterized the onset of the damage and the end of observation, the increasing trend of 12 Gy group (Fig 8A) suggests an improvement in the overall status of animals.

**Table 5:** ANOVA test results of 12 and 15 Gy compared to sham-CTRL group.

**A)**				
	**d.p.i**	**p-value**	**T-value**	**SD**
**12 Gy vs sham-CTRL**	7	p>0.05 p = 0.3151779	2.2403	1.7054_**12Gy**_ 1.2130_**shamCTRL**_
	14	**p<0.01 p = 0.0005691	4.7370	1.2873_**12Gy**_ 1.2772_**shamCTRL**_
	21	**p<0.01 p = 0.0017150	4.4110	1.4961_**12Gy**_ 0.78633_**shamCTRL**_
	30	**p<0.01 p = 0.006810	3.8764	1.3918_**12Gy**_ 0.8701_**shamCTRL**_
	40	**p<0.01 p = 0.0004174	4.9562	1.2325_**12Gy**_ 1.1076_**shamCTRL**_
**B)**				
	**d.p.i**	**p-value**	**T-value**	**SD**
**15 Gy vs sham-CTRL**	7	p>0.05 p = 0.2072	2.4225	1.4918_**15Gy**_ 1.2130_**shamCTRL**_
	14	**p<0.01 p = 1.8353e-05	5.9989	1.4257_**15Gy**_ 1.2772_**shamCTRL**_
	21	**p<0.01 p = 0017150	4.4110	1.4961_**15Gy**_ 0.78633_**shamCTRL**_
	**30**	**p<0.01 p = 1.3639e-05	6.2991	1.4159_**15Gy**_ 0.8701_**shamCTRL**_
	**40**	**p<0.01 p = 1.2712e-05	6.3274	1.3659_**15Gy**_ 1.1076_**shamCTRL**_

The differences in body weight between the two highest-dose groups (17 and 19 Gy) and sham-CTRL group were quantified and plotted in Fig 8B. In order to identify statistically significant differences in body weight loss between the two groups, simultaneous comparison Bonferroni and Holm *post-hoc* test was used. We observed a significant reduction of body weight of mice treated with the highest proton beam doses, compared to the sham-CTRL group at 7 d.p.i. (**p<0.01; T-value = 5.5213; SD_D≥17Gy_ 1.146 and SD_CTRL_ 1.213) and at 40 d.p.i. (**p<0.01; T-value = 5.6739; SD_D≥17Gy_ 1.332 and SD_CTRL_ 1.108).

Finally, skin injury-related survival proportions of the two highest dose (17 and 19 Gy) and the two lowest dose (12 and 15 Gy) groups were quantified in percentage and shown in Fig 8C. This analysis confirms a statistically significant difference between the two groups with a ****p-value < 0.0001. Although only a macroscopic and preliminary analysis was performed, our data suggest that stress caused by proton beam may not interfere with the damage response mechanisms of the organism. Surely, in-depth molecular and toxicological analysis will be required to rule out potential organ-specific injuries.

## 4. Discussion and conclusion

Proton therapy is quickly diffusing worldwide due to many advantages compared to conventional radiotherapy. The balance between efficiency and efficacy of proton therapy and the appearance of side effects is related to several issues including a correct assessment of the variation of the RBE along the BP. Currently during proton therapy treatment planning the proton RBE is assumed equal to constant and equal to 1.1 [19]. Despite this, some authors propose that proton RBE varies depending on the linear energy transfer (LET). In detail when proton energy decreases, the LET increases, especially at the end of its range where it increases dramatically [20–22]. To overcome the simplistic use of a constant RBE, further *in vivo* studies that use deterministic damage as an endpoint are required in order to determine unequivocal results transferable to clinic.

Recently, *in vivo* studies evaluated deterministic damages caused by proton therapy paying attention on the doses at which these injuries occur. In these studies both long-term endpoint, like myelopathy [4,5,11,12] where the onset happens 150–200 days post-irradiation, and early appearing injuries, like skin damages [3], have been investigated. In order to obtain relevant evidence about RBE variation, experimental configurations that mimic clinical conditions have to be used and *in vivo* experiments have to be performed [13].

This work is part of a long observational study which pursued the study of the dose at which 50% of animals develop myelopathy effects by irradiating the spinal cord in order to assess the biological impact of distal SOBP. For this purpose, animals were irradiated with a range of proton doses at spinal cord level to evaluate a possible onset of myelopathy proton beam-induced. Proton doses were calculated taking into account a previous work of Yeh Chi Lo et al. [11] where the spinal cord of mice were irradiated with a range of X-ray doses. Since the doses were high and released in only one fraction, the estimated RBE used to re-scale the X-ray doses to protons was 1.2 (6). To our knowledge, there is no study that considers the characterization of skin injury in the region of treatment used in this paper and even in Yeh Chi Lo et al. [11,12] there is no mention about any possible skin damage. Previous studies described only mild skin injury effects, like fur loss, swelling and redness and mainly at dose higher than the ones used in this paper [3,23]. As it is poorly studied, an observational analysis was realized in order to better evaluate skin injury as a proton-induced effect of different protocols of irradiation. In our view, this work wants to highlight the experimental evidence of significant skin damage effect at the doses used through an observational study and how it affects animal wellbeing.

Significant insurgence of skin injury was observed in this work from 7 days post-irradiation. Specifically, we observed that healthy mice treated using a modulated proton beam at different doses of radiation show different severity grade of skin damage, even though previous works have shown that this side effect occurs at higher doses [3,23]. According to typical burn symptoms (hyperalgesia, itching, heat), irradiated mice showed signs of suffering, such as stress, poor palatability and more restless behaviour. In detail, deep burn and necrosis of the irradiated skin tissue induced by the two highest doses used in this work (17 and 19 Gy) forced early sacrifice of some irradiated animals. On the other side, the single doses of 12 and 15 Gy guaranteed a high life index to animals which showed only early-stage ulcers. Interestingly, despite the fact that the skin injury severity induced by protons has influenced consistently the general health conditions of irradiated animals in a dose-dependent way, surviving mice showed a plateau phase or a minimum weight increase over time. Furthermore, only the body weight of the 12 Gy group maintained a positive trend, regardless of irradiation, until the end of the observation. In preclinical studies, the use of ED50 may help to predict the threshold dose beyond which the severity of the skin lesions could critically affect the health condition of animals, helping to avoid its sacrifice before reaching long term endpoint, such as senescence, myelopathy or metabolic dysfunctions. Thus, the ED50 values obtained in this work represent an important attainment that could be used to assess a preclinical in vivo RBE if similar results using a reference radiation become available.

In conclusion, although this study would need further investigation to specify in detail the variation of the RBE along a Bragg peak, also by using data from x-ray (i.e. 250 keV) radiation as reference, it suggests to analyse the side effects thoroughly, especially the early ones, such as skin damage laying the bases for more dedicated preclinical research.
